# Cerebrospinal fluid penetration of very high-dose meropenem: a case report

**DOI:** 10.1186/s12941-018-0299-0

**Published:** 2018-12-29

**Authors:** Thomas Kerz, Friederike D. von Loewenich, Jason Roberts, Axel Neulen, Florian Ringel

**Affiliations:** 1 Department of Neurosurgery, University Medical Center, Langenbeckstr. 1, 55131 Mainz, Germany; 2 Department of Medical Microbiology and Hygiene, University Medical Center, Mainz, Germany; 3 University of Queensland Centre for Clinical Research, Faculty of Medicine, The University of Queensland, Brisbane, QLD Australia; 4 Department of Intensive Care Medicine, Royal Brisbane and Women’s Hospital, Brisbane, Australia; 5 Pharmacy Department, Royal Brisbane and Women’s Hospital, Brisbane, Australia; 6 Centre for Translational Anti-infective Pharmacodynamics, School of Pharmacy, The University of Queensland, Brisbane, QLD Australia

**Keywords:** Meropenem, Cerebrospinal fluid, *Acinetobacter baumannii*, Carbapenem resistance, Meningitis, Case report

## Abstract

**Background:**

Standard dosing of meropenem (2 g t.i.d.) produces CSF concentrations of only 1–2 mg/L which is inferior to the clinical breakpoint for most Gram-negative bacteria. There is therefore concern that dosing must be increased in order to achieve therapeutic CSF concentrations for bacteria with susceptibility close to clinical breakpoints. Yet, the effects of high-dose meropenem on CSF concentrations are not well described in literature. We therefore determined meropenem CSF-levels in a patient who was treated with 15 g/day of meropenem.

**Case presentation:**

Our patient suffered from a brain trauma and an external ventricular drainage was implanted. Later, a carbapenemase-producing *Acinetobacter baumannii* (OXA-23, NDM-1) was isolated from blood cultures and CSF. The MIC for meropenem was > 32 mg/L (R), and we opted for a combination therapy of meropenem, colistin and fosfomycin. Meropenem was given at an unusual high-dose (15 g/day) with the aim of achieving high CSF concentrations. CSF concentrations peaked at 64 mg/L. Yet, the patient succumbed to an intracranial bleed into a preexisting cerebral contusion.

**Conclusions:**

High-dose meropenem can achieve CSF levels largely superior to those achieved with commonly recommended dosing regimens. Though our patient succumbed to an intracranial bleed which could be regarded as a severe adverse event, we suggest that meropenem dosing can be increased when pathogens with increased MICs are found in the CSF. More in vivo data are however needed to determine the safety of high-dose meropenem.

## Background

Meropenem is a frequently used antimicrobial for post-neurosurgical meningitis/ventriculitis [1]. It has been shown to be clinically effective [1], but its penetration into the cerebrospinal fluid (CSF) is not well characterized. The median penetration rate ranges from 9 to 39% with high inter-individual variation [2, 3]. The efficacy of β-lactams such as meropenem depends on the percentage of the time that the unbound fraction remains above the minimum inhibitory concentration (MIC) of the microorganism. According to animal models the target for Gram-negative organisms in blood is 40–50% > fT > MIC [4] and an important determinant for patient outcome [5]. For CSF, the precise pharmacodynamic target is still subject of debate. For beta-lactam antibiotics, time above the minimal bactericidal concentration (t > MBC) possibly determines the rate of bactericidal activity in CSF, and a t > MBC for more than 50% of the dosing interval has been recommended [6]. Yet, no major distinction between MIC and MBC was found in several other studies [6–8], so it is open to debate if MIC or MBC need to be targeted. Meningeal inflammation facilitates the penetration of β-lactams into the CNS but most nosocomial CSF infections will only result in minimal-to-mild disturbances in the blood–CSF barrier [9].

The usual regimen of 2 g meropenem every 8 h results in CSF concentrations of 1–2 mg/L which is lower than the clinical breakpoint for susceptibility for most Gram-negative organisms [10]. Therefore, this regimen might be inadequate for the treatment of CNS infections leading to prolonged courses of treatment as well as patient morbidity and/or mortality. Continuous infusion of β-lactams has also been applied to ensure adequate plasma concentrations [11]. However, data on the efficacy of this regimen in meningitis/ventriculitis are scarce. Measurement of meropenem CSF concentrations has been recommended to ensure clinical efficacy in critically ill patients [2], although CSF concentrations might not exactly mirror brain tissue concentrations. We report on a high-dose meropenem regimen in a patient with a carbapenem-resistant *Acinetobacter baumannii* brain abscess where relevant CSF concentrations could be attained with very high-dose meropenem. To our knowledge, this is the first report on meropenem CSF concentration with such a high-dose regimen.

## Case presentation

Our patient developed an intracranial abscess after placement of an external ventricular drain because of a severe brain trauma. CSF results are given in Table 1.

**Table 1:** CSF-results during days 1–33

Days	1	5	8	12	15	19	19	23	26	29	33	33	Unit	Normal
Leukocyte count (CSF)	108	192	150	88	790	3190	105	274	225	72	1470	540	/μL	< 5
Erythrocyte count (CSF)	11	300	3165	1452	9590	7890	5925	387	3100	997	34,500	13,630	/μL	0
Lactate (CSF)	6.5	9.7	8.8	6	7.4	9.5	5.8	5.4	5.7	5.4	7.7	7.3	mmol/dL	1.7–2.6
Total-protein (CSF)	631.8	146.7	145.5	101.6	231.1	168.6	111.6	113.2	197.8	91.1	2956	1824.6	mg/dL	15–40

**Fig. 1:**
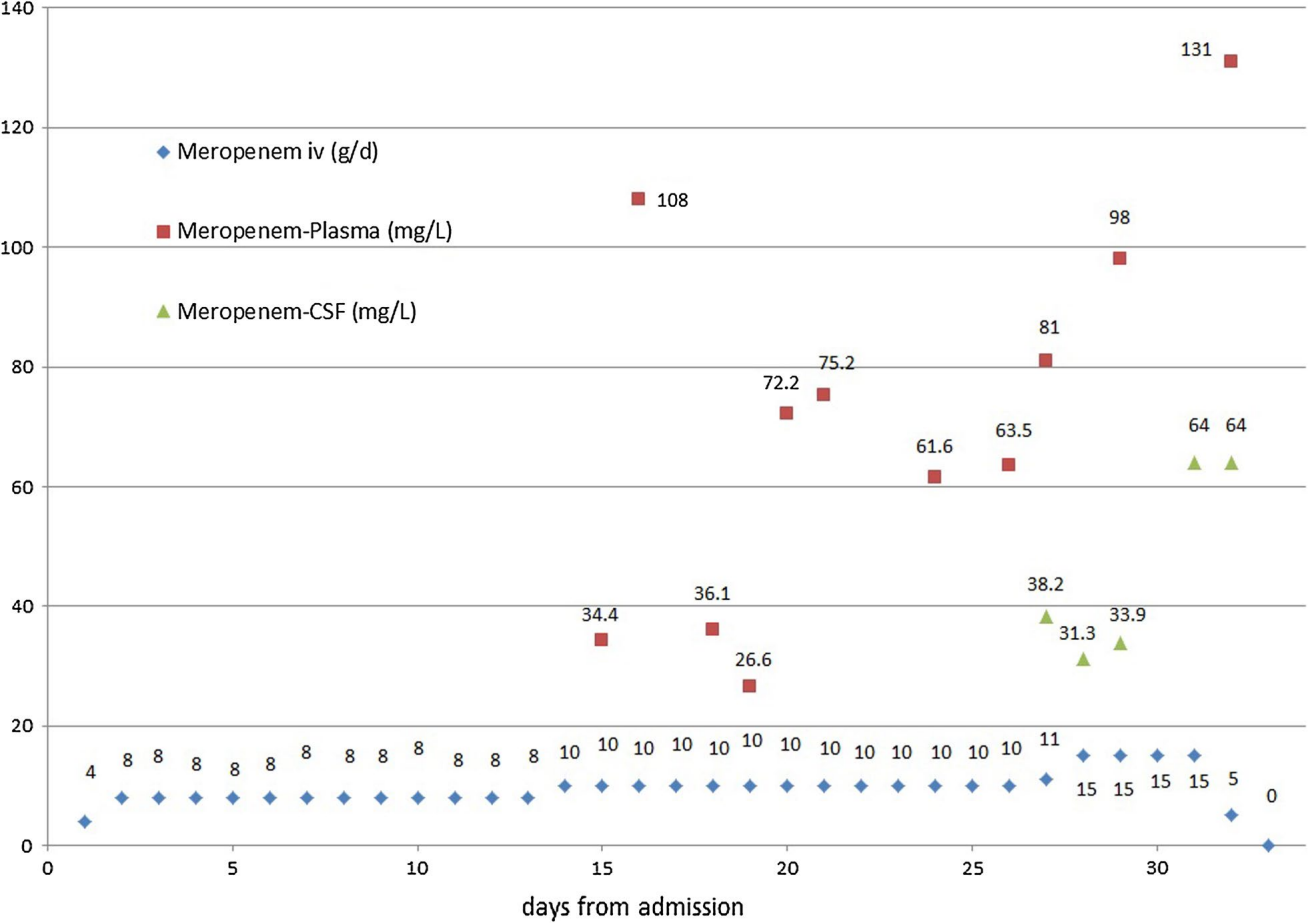
Meropenem doses in g (blue diamonds) during i.v. treatment from day 0 to day 33. Meropenem plasma (red square) and CSF (green triangle) levels in mg/L, measured by HPLC-UV detection. X-axis in days, y-axis g/day resp. mg/L

A carbapenemase-producing *A. baumannii* (OXA-23, NDM-1) was isolated from blood cultures and CSF. The MIC for meropenem was determined using a gradient test (MIC-test strip^®^, Liofilochem, Roseto degli Abruzzi, Italy) and for colistin via commercial broth microdilution (Micronaut-S^®^, Merlin Diagnostika, Bornheim-Hersel, Germany). European Committee on Antimicrobial Susceptibility Testing (EUCAST) breakpoints were applied. The MIC for meropenem was > 32 mg/L (R) and for colistin ≤ 1 mg/L (S). Colistin, fosfomycin and meropenem were given because clinical data point to improved outcomes even in carbapenem-resistant A*. baumannii* infections using combination therapy [12]. Meropenem was commenced with 2 g every 6 h, and then increased initially to 10 g/day and then to 15 g/day in doses of 2 g given over 3 h with a single dose of 1 g/3 h once daily, therefore allowing continuous meropenem infusion avoiding high peaks and low troughs. Meropenem concentrations in plasma and CSF were determined once daily during a 2 g dosing interval by HPLC–UV detection (Fig. 1). The liquid chromatographic system (Agilent 1200) consisted of an HPLC quaternary pump, a thermostated oven, a thermostated autosampler, and an UV-diode array detector. Separation was achieved at 45 °C utilizing a Waters X-Bridge analytical column (C18; 4.6 × 100 mm, 3.5 µm). The mobile phase consisted of 20 mM potassium dihydrogen phosphate pH 2.6 (mobile phase A) and 20 mM potassium dihydrogen phosphate pH 2.6 in 50% acetonitrile (mobile phase B). Flow rate was 1.5 mL/min and injection volume was 10 µL. The autosampler temperature was kept at + 4 °C. UV-absorbance was monitored at 300 nm. The gradient started at 12% B and increased to 17% B over 5 min and then to 50% B over 3 min. After a washing step at 95% B for 0.5 min the column was re-equilibrated at 12% B for 3 min. Data analysis was performed using Agilent ChemStation software. Proteins were precipitated by adding 400 µL Internal Standard solution (40 mg/L cefotaxime in acetonitrile) to 200 µL serum. After vortexing precipitated proteins were separated by centrifugation. The supernatant was mixed with 3 mL of dichloromethane, vigorously vortexed and centrifuged. An aliquot of the upper aqueous phase was transferred to an autosampler vial for chromatographic analysis.

Since creatinine clearance has been found to be an important covariate of meropenem pharmacokinetics [13], we monitored renal function closely. The calculated glomerular filtration rate (CKD-EPI) was ≥ 50 mL/min/1.73 m^2^ initially but decreased to 23–40 mL/min/1.73 m^2^ after day 26, most probably due to the concomitant infusion of colistin. CSF meropenem concentrations varied between 31 and 38 mg/L when 10 g/day were given over a time period of 12 days. The increased dosage of 15 g/day for 3 days resulted in a peak CSF concentration of 64 mg/L (Fig. 1). The CSF concentration of meropenem was 34–49% of the serum level. Frequent vomiting and a gastroparesis were noted as side effects whereas there was no effect on hematological or liver parameters. No assessment of central nervous side effects was possible due to the patient being in coma. Unfortunately, a massive intracranial hemorrhage into the area of contusion occurred and the patient died on day 33.

## Discussion and conclusions

Commonly recommended meropenem doses for meningitis/ventriculitis are 2 g every 8 h [14]. Yet, meropenem penetration into the brain is subject to large inter-individual variation and median penetration ratios of as low as 9% have been found [2]. Further, it is unclear which percentage of meropenem in the CSF is in the unbound active state. Therefore, the CSF concentrations of meropenem (6 g/24 h) might not be sufficient to kill bacteria with increased MIC (> 2 mg/L) [15]. An augmented renal clearance in up to two-thirds of intensive care patients possibly also contributes to under-dosing of antibiotics [16].

A prolonged 4-h meropenem infusion of 2 g every 8 h leads to a probability of a target attainment rate of > 90% for most Gram-negative organisms in the blood compartment, when the MIC of a Gram-negative organism is ≤ 8 mg/L. Monte Carlo simulations suggested that, to reach this with the same dosing regimen in CSF, the MIC has to be as low as ≤ 0.5 mg/L [17]. In a pharmacokinetic model, 5 g meropenem every 8 h were needed to achieve CSF trough concentrations of > 2 mg/L in 95.1% of simulated patients [2]. Therefore, even the meropenem dose of 2 g every 8 h probably fails to achieve a reasonable target attainment rate in CSF, especially in infections with Gram-negative organisms with MICs in the intermediate or resistant range. In our patient, the use of high dose-meropenem resulted in CSF concentrations largely superior to those reported in the literature when standard dosing regimens are given. According to our results, CSF pathogens with increased meropenem MICs might be treated successfully with high-doses of meropenem, and our case suggests that an increased meropenem dosing should strongly be considered in such cases. Considering the highly variable kinetics, therapeutic drug monitoring in both serum and CSF seems indispensable to assure achievement of therapeutic drug concentrations. It is, however, unclear if the fatal bleeding was related to the high dose of meropenem. Further data are therefore necessary to determine the safety of such treatment and if dosing recommendations should be changed.

## Abbreviations

CKD-EPI: Chronic Kidney Disease Epidemiology Collaboration
CNS: central nervous system
CSF: cerebrospinal fluid
EUCAST: European Committee on Antimicrobial Susceptibility Testing
fT: fraction of time
HPLC–UV: high performance liquid chromatography–ultraviolet
MIC: minimal inhibitory concentration
NDM 1: New Delhi metallo-beta-lactamase 1
OXA-23: oxacillinase-23
